# Recent improvements to the selectivity of extraction-based optical ion sensors

**DOI:** 10.1039/d1cc06636f

**Published:** 2022-02-16

**Authors:** Kye J. Robinson, Yoshiki Soda, Eric Bakker

**Affiliations:** Department of Inorganic, Analytical Chemistry University of Geneva Quai Ernest-Ansermet 30 1211 Geneva Switzerland Eric.Bakker@unige.ch

## Abstract

Optical sensors continue to demonstrate tremendous potential across a wide range of applications due to their high versatility and low cost. This feature article will focus on a number of recent advances made in improving the performance of extraction-based optical ion sensors within our group. This includes the progress of anchored solvatochromic transduction to provide pH and sample volume independent optical responses in nanoemulsion-based sensors. A recent breakthough is in polyion sensing in biological fluids that uses a novel indirect transduction mechanism that significantly improves the selectivity of dinonylnaphthalenesulfonate-based protamine sensors and its potential applications beyond polyion sensing. The role of particle stabilizers in relation to the response of emulsified sensors is shown to be important. Current challenges in the field and possible opportunities are also discussed.

## Abstract 2

Selectivity remains a constant challenge in the development of optical extraction-based sensors. Fortunately, there are several mechanistic and compositional changes with the potential to improve selectivity without developing new ionophores.

## Graphical abstract

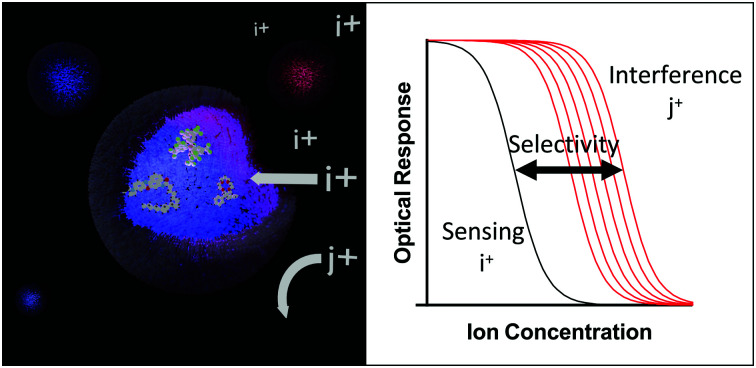

## Optical ion sensors

Ion sensing has long been a core focus of analytical chemistry as the concentrations of ionic species are critical for the proper function of ecological systems, homeostasis of biological organisms and ensuring quality standards of industrial materials. One of the largest advancements in this field was the development of ion-selective electrodes (ISEs), which have become widely used across a number of disciplines.^1^ The excellent performance of these sensors originates from the properties of the underlying ion-selective membrane. These membranes generally consist of three main components: a hydrophobic matrix, which provides a large thermodynamic barrier towards entry of highly charged species in addition to structural support; ionophores,^2^ ligands selective for a target ion; and an ion exchanger, lipophilic salts used to maintain the total ion quantity in the membrane through electroneutrality. ISEs have long shown very promising performance in a variety of complex samples such as biological fluids, sea water, beverages *etc.* due to their high selectivity and sensitivity.^3^

Ion-selective optodes, or ISOs, have emerged as a powerful variant of ion-selective membranes with 10,578 available publications with in the Web of Science Core Collection (search terms: ion selective optode* OR optical ion sens*).^4,5^ These sensors provide an optical readout obtainable using a spectrometer, camera or even the human eye.^6^ Unlike traditional ISEs, an optical readout allows the decoupling of sensor and detector enabling measurements through optically transparent barriers for example cell membranes^7^ while also lending itself to 2D or even 3D chemical imaging.^8,9^

**Fig. 1:**
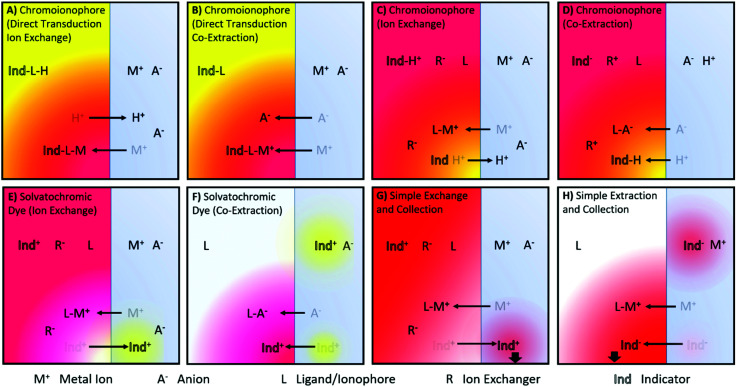
Illustrations of reported ion sensing mechanisms. Note that mechanisms depicted with a cationic dye have also been investigated for anionic dyes for analytes of the opposite charge. (A and B) Direct transduction using a analyte specific chromoionophore with co-extraction of an anion or a chromoionophore which deprotonates^10,11^ (C and D) Hydrogen Chromoionophore-based sensors operating through either exchange or co-extraction depending on the charge of the analyte.^12^ (E and F) Solvatochromic dye-based sensor signaling a polarity shift as it moves from the hydrophobic sensing matrix to an aqueous environment^13^ or vice versa^14^ (G) Dye undergoing exchange and changing the absorbance of the sample solution.^6^ (H) Dye can also be co-extracted into an organic phase, the absorbance of which can then be measured.^15^

A number of ISO sensing mechanisms are displayed in Fig. 1. As shown in the Figure, ISOs in addition to the components found in ISEs, require an optical transducer. This most commonly takes the form of a chromoionophore that changes optical properties following target binding.

Charlton and coworkers from Ames Research Laboratories were the first to introduce ion exchange-based optical transduction by a lipophilic dye confined to the sensing phase that can bind to a hydrogen ion, Fig. 1A.^16^

Subsequently, Morf and coworkers suggested the use of hydrogen ion-selective chromoionophores (pH-chromoionophores) in optode sensors and an ion exchange mechanism for optical signal transduction to develop reversible sensors that has become the most widespread mechanism used for ISOs, Fig. 1C.^12^ This approach circumvents the need to produce a highly selective chromoionophore for every analyte that also changes its optical response upon binding, which is an extremely research-intensive exercise. Hydrogen ion-selective chromoionophores are typically coupled with an ionophore pulled from the highly selective pool of ionophores developed for ISEs. This allows the selective extraction of the target ion into the organic sensing phase by the exchange with a chromoionophore-bound hydrogen ion. The latter changes the protonation state-dependent absorbance/fluorescence spectra and thereby enables the optical quantification of ions. For the detection of anions, a similar approach may be applied. Instead of the positively charge analyte displacing a chromoionophore-bound hydrogen ion, a hydrogen ion in solution is co-extracted into the organic phase with the analyte, Fig. 1D. Solvatochromic dye based sensors (Fig. 1E and F) operate in a similar fashion except instead a hydrogen ion undergoing exchange/co-extraction the dye itself is transported between phases. The dye then optically signals the polarity of the environment in which it is contained. Both chromoionophore and solvatochromic mechanisms lend themselves to either particle or film-based sensors. In contrast, mechanisms utilising dyes with static optical spectra (Fig. 1G and H) are generally only employed in films due to the need to separate the sample and sensing phase for accurate measurements and have found applications in capillary or dipstick type sensors.^6^

The detection range of these sensors is a function of ionophore binding constant and equilibrium of the optical transducer, and as such is tuneable using a number of parameters such as pH in the case chromoionphore-based sensors or transducer lipophilicity in the case of solvatochromic sensors.^17,18^ When using substantial amounts of high affinity ionophore it is also possible for the sensor to extract a majority of the analyte from the sample solution. In this case the response is said to operate in an exhaustive mode rather than equilibrium mode and the response range will be shifted to higher analyte activities.^19^ Previous work has compared chromoionophore-based Pb^2+^ ISOs to equivalent ISEs and found that bulk optodes have a much lower detection limit than their classical counterparts (7.9 × 10^−14^ vs 6.3 × 10^−10^ M Pb^2+^) while maintaining typical response ranges (5 orders and greater than 4 orders of magnitude for ISOs and ISEs respectively) but it should be noted that this lower detection limit comes with extremely long response times.^20^ In this work, for concentrations below 3.2 × 10^−12^ M in EDTA buffer, response times greater than 60 h were reported. This was in part attributed to the slow decomplexation with EDTA. Later studies achieved similar detection limits in optode films but with response times of 50 s for 5.3 × 10^−13^ M with a linear response range of 1.0 × 10^−12^ M – 8.6 × 10^−4^ M.^21^ For many practical applications however, selectivity rather than detection limit is the biggest hurdle with sample matrices often containing several ions with similar physical properties.

In principle, ISOs require derivatization of reagents in the sensing phase, such as ionophore–ion complexation and protonation/deprotonation of dyes, in contrast to typical ISE membranes that do not change inner chemical composition during signal transduction.^1^ In this sense, ISOs may more greatly influence the sample composition than ISEs, however this provides more flexibility in signal transduction mechanism, which has led to the invention of varied mechanisms for optical signal transduction. In addition, ISOs are liberated from an electrode body, giving greater diversity of sensor configuration and shape.

The field of optical ion sensing now continues to push the limitations of selectivity, developing new ionophores and binding agents with the ever-improving tools at our disposal. There are, however, several ways in which selectivity can be improved without developing new ligands or ionophores. By taking advantage of the analyte nature and mechanistic advances, it is possible to make significant improvements to selectivity obtained using the same currently available ionophores. This article will focus on some of the recent advances made by our group in this area.

## The performance gap between membrane and particle type sensors

The theory describing the ISO response was studied in the '90s and originated from that describing ISEs^5,22^ as the principal difference in the basic membrane components of these two systems is the presence of a chromoionophore as indicator. Below is one of the most typical ion exchange sensing reactions by ISOs, written for the detection of a monovalent ion M^+^:^22^

1 M^+^(aq) + *n*L(org) + IndH^+^(org) ⇄ ML^+^_*n*_(org) + Ind(org) + H^+^(aq)

Where L, Ind, ML^+^_*n*_, IndH^+^ and H^+^ represent ionophore, deprotonated chromoionophore, ion–ionophore complex, protonated chromoionophore and the hydrogen ion, respectively and *n* is the stoichiometry of the ion–ionophore complex (M^+^ : L = 1 : *n*). The phase labels are for the aqueous (aq) and organic sensing phase (org), respectively. The equilibrium constant *K*^ILn^_exch_ for this ion exchange is expressed as eqn (1):^22^2
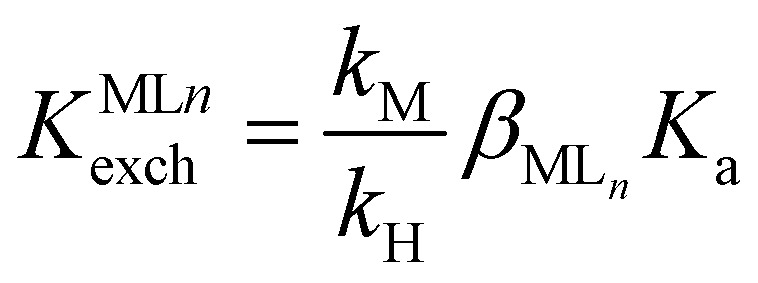
3
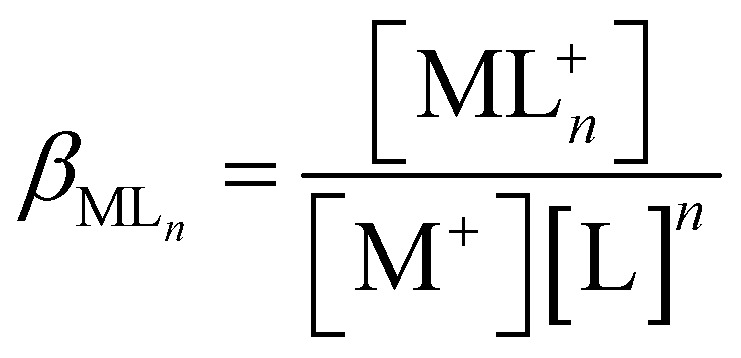
Where the ratio 
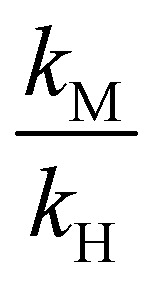
 is a function of the relative lipophilicities of the two ions,^22,23^*β* is the overall complex formation constant (eqn (3)) and *K*_a_ is the acid dissociation constant. Species in square brackets denote concentrations in the organic sensing phase.

For monovalent ions, the selectivitiy coefficient (*K*^opt^_I,J_) is quite simply the ratio of two such ion exchange constants determined for a primary and an interfering ion, denoted as I^+^ and J^+^, respectively, which is in turn directly dependent on the lipophilicity of the two ions and the complex formation constants in the sensing film:4
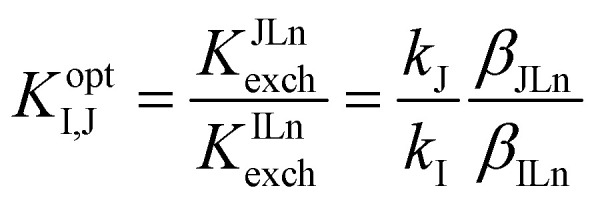


**Chart 1:**
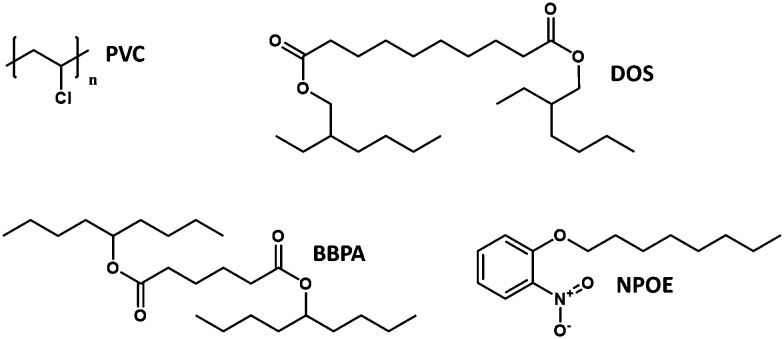
Structures of commonly used optode matrix materials represented in Table 1. Matrices containing PVC are generally among the most hydrophobic resulting in both the highest energy barrier to ion entry and the highest binding constants of incorporated ionophores.

The relationship is more complex for species of different valency and complex stoichiometry.^22,23^ Complex formation constants are not of a fixed value for a given pair of ionophore and ion because the value also depends on the matrix in which they are contained. This principle also applies to chromoionophores. Table 1 summarizes a limited set of observed stability constants of ionophores and p*K*_a_ values of chromoionophores in various membrane matrices together with particle-type sensors in order to demonstrate how ionophore binding constants can change, based not only on sensor composition, but sensor type. In this article, the stability constants obtained *via* optical methods are shown while various electrochemical methods such as sandwich membrane^24,25^ and voltammetry-based assays^26^ are not considered here. As seen in Table 1 stability constants of the ionophore–ion complex and p*K*_a_ values vary significantly with matrix. This is because each matrix exhibits a distinct hydrophilicity and solvent polarity that dictates the stabilization of ionic species in the membrane, Chart 1. For example, the stability constants of ionophores and the p*K*_a_ values of electrically neutral chromoionophores tend to be lower as the matrix is more hydrophilic. Because the selectivity is highly dependent on these values it follows that sensor selectivity is closely tied to matrix properties. While gathering data for the above table it also became apparent how rarely both binding constants and chromoionophore p*K*_a_ values are reported in the same article. More widespread reporting of these values in parallel could provide an opportunity to construct larger comparative data sets useful for interrogating matrix effects of the ionophores themselves in addition to other potentially unexplored effects.

**Table 1:** Select stability constant of several ionophores and chromoionophore p*K*_a_ values of ISOs various membrane and nanoparticle compositions

Sensor type	Matrix	Ionophore/ion	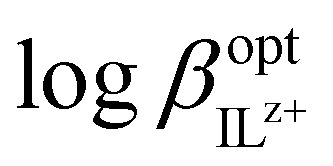	Transducer	p*K*_a_
Membrane	PVC/DOS (1 : 2)	Valinomycin/K^+^	9.3^27^	CH1	11.4,^28^ 12.0^29,30^
		Calcium ionophore II/Ca^2+^	23.8^27^	CH2	9.16,^28^ 10.2^29,30^
				CH3	8.00,^28^ 13.4^29,30^
Membrane	PVC/NPOE (1 : 2)	Chromoionophore Only		CH1	14.8^28^
				CH2	12.3^28^
				CH3	9.59^28^
Membrane	PVC/BBPA	Sodium ionophore V/Na^+^	7.4,^27^ 7. 6^27^		
Nanoparticle	PVC/DOS (1 : 2)	Valinomycin/K^+^	5.93^31^	CH1	7.84,^31^ 8.8 (6,8.5,10.5)^a^^ ^^32^
				CH3	5.5 (3.3,5.4,7.7)^a^^ ^^32^
Nanoparticle	DOS only	Chromoionophore Only		CH1	8.8 (9.9,7.8)^a^^ ^^33^
				CH3	10.2 (11.8,9.8,7.2)^a^^ ^^33^
Nanoparticle	DOS only	Sodium ionophore X/Na^+^	∼4,^34^ 4.8^35^	CH1	5.9^34^
		Valinomycin/K^+^	6.4^35^	CH1	
		Sodium ionophore X/Na^+^	4.7^35^	SD	
		Valinomycin/K^+^	6.1^35^	SD	
		Calcium ionophore IV/Ca^2+^	9.9^35^	SD	
		Tridodecylamine/H^+^	8.3^35^	SD	

a Multiple p*K*_a_ values fit.

**Fig. 2:**
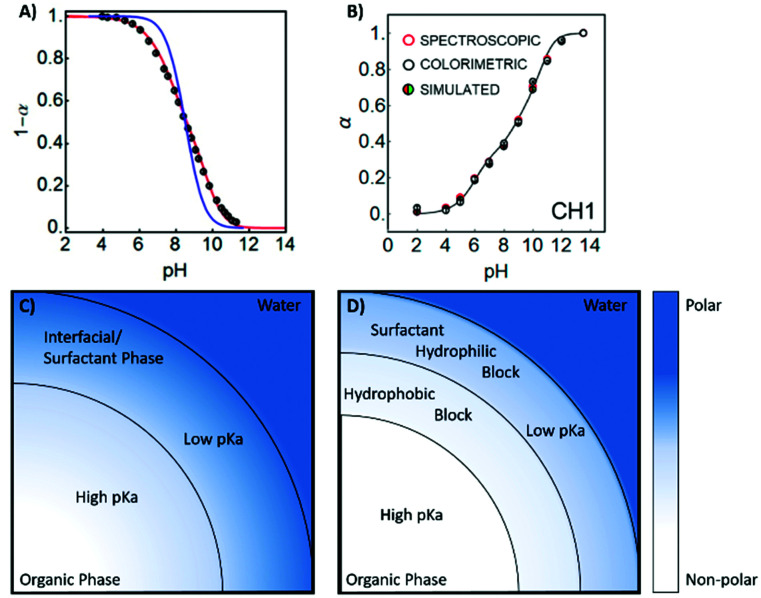
(a) Reproduced from Xie *et al.*^33^ Multiple p*K*_a_s fitted (red) *versus* a single p*K*_a_ (blue) in a nanosensor system (b) highly distinct p*K*_a_s in a surfactant stabilized nanosensor system from Soda *et al.*^32^ (c and d) internal polarity of emulsion based optodes.

The substrate matrix polarity is not the only factor affecting the p*K*_a_ values and stability constants. From Table 1 it becomes immediately apparent that in addition to the effects of the sensing matrix material the sensor type (film or nanoparticle) has a dramatic influence on the apparent binding constants. The move to emulsion-based sensors, which are particularly attractive due to their rapid response times, was found to decrease sensor binding constants often by several orders of magnitude,^35^ meaning that the energy difference between ionophore bound ions and so called “free ions” is reduced within the sensing matrix. The result is a much higher concentration of free interfering ions in the sensing phase, representing a significant challenge for sensor selectivity. The other related effect is a decrease of the chromoionophore p*K*_a_, which indicates an increased charge stabilisation. Work carried out by Xiaojiang *et al.* provides evidence that this stabilisation varies within a particle system, resulting in multiple apparent overlapping p*K*_a_ values.^33^ This observation might potentially be attributed to particle-to-particle variation, but it is more likely to be due to a polarity gradient within the particle, see Fig. 2.

More recent work in our laboratory has shown that rather than a continuous gradient, one possible explanation of earlier results, these different p*K*_a_s can become quite defined (Fig. 2b) suggesting regions of distinct polarity.

## Recovering lost selectivity between films and particles

When moving from sensing films to nanoscale particles a significant performance decrease was observed but little was done to address these problems besides limited cases when particles can be formed in the absence of surfactant.^36^ Particle based sensors however, often require stabilising surfactants or coatings and these same surfactants had been previously shown to negatively impact performance of analogous ion-selective electrodes^37,38^ and also blamed for poor optode sensing characteristics.^36^ Despite this, these surfactants are still commonly employed.^39–42^ We have recently shown that zwitterionic surfactants have the potential to stabilize particle-based sensors without negatively impacting binding constants and selectivity.^43^

**Fig. 3:**
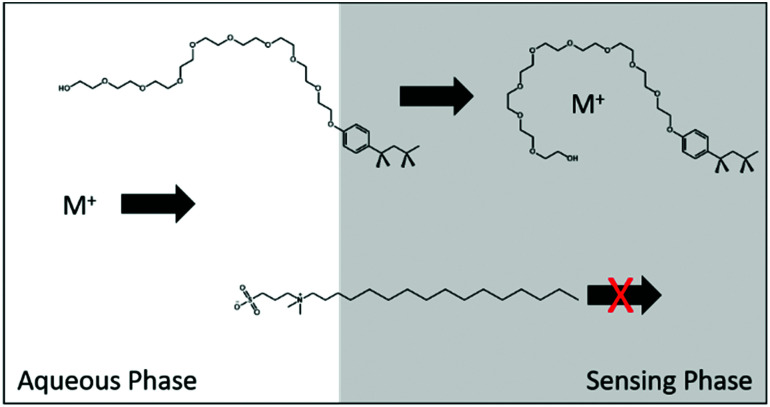
Transition of non-ionic surfactant (Trition X-100) into an organic phase stabilizing a metal ion non-specifically in contrast to a zwitterionic sulfobetaine-based surfactant, which does not undergo this interaction.

Electrically neutral surfactants are often capable of partitioning into the organic phase where they may act as a non-specific ionophore. Ethylene glycol based non-ionic surfactants are particularly prone to this behaviour, see Fig. 3. This can be described by the extraction of surfactant into the sensing phase where it complexes with the free ions as follows, shown again for monovalent ions:^43^

5 S(aq) ⇄ S(org)

6 M^+^(org) + *n*S(org) ⇄ SM^+^_n_(org)

**Fig. 4:**
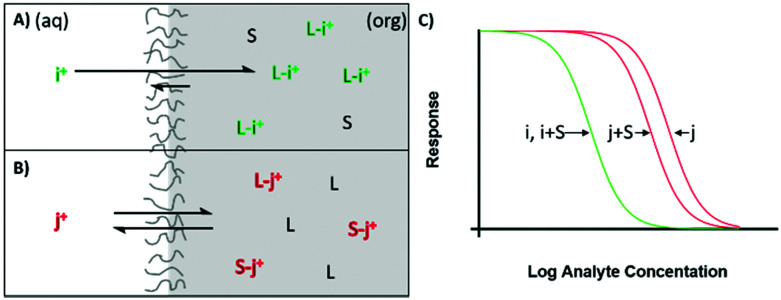
Extraction of target (i) or interfering ion (j) into an ion selective optode containing a selective ionophore (L). The total amount of ion that can be transferred is limited by the available ion exchanger not depicted here. (A) The target ion is readily extracted into the membrane, in the presence of surfactant a quality ionophore will still be preferred due to much higher binding constants. (B) As the interfering ion binds only weakly to the ionophore it is excluded, surfactant may have comparable or stronger binding constants to interfering ions than the ionophore, resulting in deteriorated selectivity. (C) Response of target and interfering ion with and without surfactant.

Therefore, the selectivity of the sensor is not only dictated by the lipophilicity of ions and ionophore selectivity, but also the selectivity of surfactant used to stabilize the particle, to which the above mentioned poor performance of particle based sensor can be ascribed. When an excellent ionophore exhibiting strong binding is present in a particle, the effect of surfactant partitioning will be negligible to the sensing of the primary ion because the ability of the ionophore to form a complex with the primary ion greatly exceeds that of surfactant. In contrast, partitioned surfactant has a greater opportunity to form complexes with interfering ions, as ionophores have much lower binding constants towards them, Fig. 4.^43^ Thus surfactant complexation only significantly alters the extraction of interfering ions and the selectivity is worsened compared to that of bulk optodes.

The stability constant will be similar between interfering and primary ions due to poor selectivity of surfactant species as a ligand. In our recent work we estimated the logarithmic binding constants for Brij-35 and Triton X-100 to be 6.2 and 6.5 towards potassium and 5.5 and 6.3 towards sodium, respectively. This compares to the potassium ionophore valinomycin with logarithmic binding constants of 10.2 towards potassium and 5.36 for sodium.^43^

In contrast to the above surfactants, zwitterionic surfactants seem to be either better at remaining at the particle interface due to their highly charged head groups or do not stabilise free ions in the organic phase to the same degree all while having an overall neutral charge. Based on this evidence it is also reasonable to attribute changes in chromoionophore p*K*_a_ to surfactant interactions.

Using the zwitterionic surfactant (3-(*N*,*N*-dimethlpalmitylammonio)propanesulfonate, SB16) it was possible to obtain a doubling of the selectivity, as well as achieving improvements to the effective binding constants by several orders of magnitude 
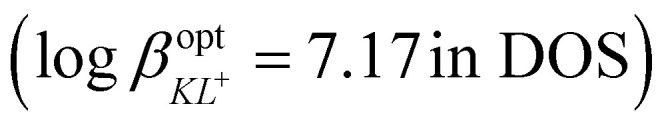
 over three non-ionic surfactants: Pluronic F127 (5.2), Brij-35 (4.9) and Trition X-100 (5.3) in a potassium selective emulsion-based optode. This difference became even more pronounced in more hydrophobic matrices based on plasticized PVC (SB16, >8; F127, 4.38). The improvements to selectivity may be even more significant when the major interfering ions are slightly more lipophilic, for example for sodium sensing with potassium as an interfering ion. This work demonstrates the importance of careful surfactant selection. However, although this surfactant was particularly useful for these types of sensors careful consideration should be given to each application, as discussed below with regards to our recent work on polyion sensing.

## Moving from chromoionophores to anchored solvatochromic dyes

One of the major limitations of hydrogen chromoionophore-based sensors has been the pH dependence of the transducer. In very specific circumstances such as in CO_2_ sensing, the analyte concentration is thermodynamically tied to pH and this pH dependence is cancelled out.^44^ However, in most cases this pH dependence necessitates the addition of a buffer to the sample, which is undesired.

Interestingly, the earliest optodes did not utilise pH-sensitive indicators such as chromoionophores but were pH independent. They were potassium-selective test strips based on the coextraction of K^+^ and an anionic dye, erythrosine B, into a film containing valinomycin.^15^ Wolfbeis and coworkers then used potential sensitive dyes to optically indicate the phase boundary potential.^45^ In that paper, the fluorescence signal change is explained to be due to the potential change at the membrane/sample phase boundary upon recognition of K^+^. However, today one would rather describe this effect as ion exchange between K^+^ and the positively charged dye that is solvatochromic.

This formed the basis for subsequent work on pH-independent microparticle sensors developed by the same group.^46^ These sensors were based on coextraction of anionic species and positively charged solvatochromic dyes that were sufficiently hydrophobic as to not experience complete extraction into the aqueous sample phase. Bakker and coworkers also developed pH independent sensors based on ion exchange as shown in Fig. 1F.^13,35,47^ These nanoparticle-based sensors rely on complete transfer of solvatochromic dye upon ion exchange or coextraction (Fig. 1E and F). This class of sensor using relatively hydrophilic dye is useful for a broad range of applications including not only typical determinations of cations and anions based on ion exchange or coextraction but also to complexometric titrations,^47^ the determination of stability constants^35^ and exhaustive sensing^6^ with robust theory. It demonstrated similar versatility to chromoionophore-based sensors but with pH-independency. This approach was also useful in that both dye lipophilicity and doped concentration could be altered in order to control exchange equilibrium. Unfortunately, however, this two-phase extraction principle gives signals that depend on sample volume.

**Fig. 5:**
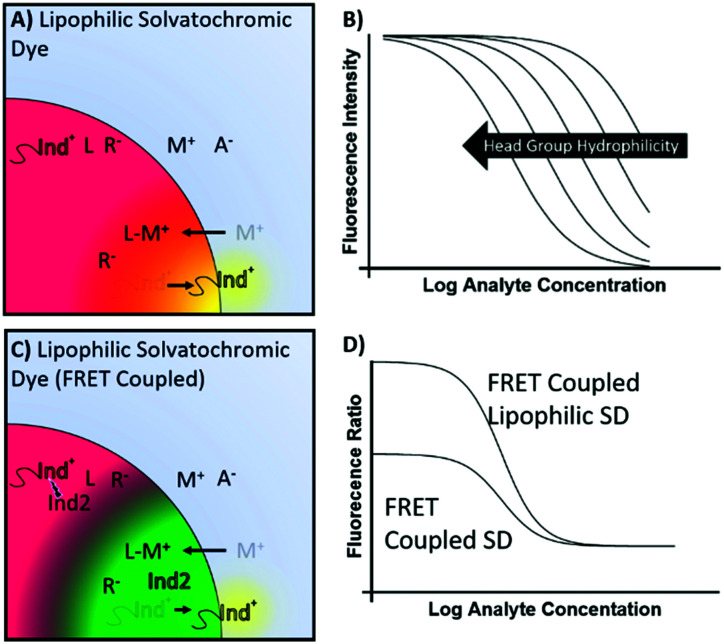
(A) Anchored solvatochromic dyes provide a pH and volume independent response. (B) Response range can be modulated by altering the hydrophobicity of the solvatochromic dye head group. (C) Solvatochromic dyes can also be paired to another FRET dye in order to provide a larger spectral shift and enable a ratiometric fluorescence readout. (D) FRET interactions with more lipophilic dyes may amplify optical response allowing more precise ratiometric measurements.

A former approach by Wolfbeis^46^ that employed a hydrophobic solvatochromic dye that transitions between the particle surface and bulk phase, also became the basis for surface accumulation type nanoparticle optodes developed by Xie and coworkers.^48^ Because the sensing mechanism of these particles involves the partitioning of solvatochromic dye between the bulk and the surface of the sensing phase, this class of sensors is best suited to micro or nanoparticle-based formats. While Wolfbeis trapped sensing droplets within a hydrophilic membrane to maximise surface area, our group showed that nanoemulsion based sensors can be used without the need for embedding in a matrix.^36^ Similarly to the equilibrium exchange modulation obtained by tuning the hydrophilicity of dyes undergoing exchange, response modulation was recently also shown to be achievable using lipophilic surface accumulation dyes through modulation of the lipophilicity of the solvatochromic head group. This allows one to tune the sensing response range (Fig. 5A and B) without inducing sample volume dependent effects.^18,49^ Of course, tuning the response range by changing the head group hydrophilicity will have its practical limits dictated by the need to maintain adequate selectivity and response range. The coupling of lipophilic solvatochromic dyes with a second dye capable of acting as a Förster resonance energy transfer (FRET) pair has also shown significant utility in the field of ion sensing, (Fig. 5C).^49^ The addition of a second dye allows for ratiometric fluorescence measurements, helping to alleviate many of the difficulties in the quantification of fluorescence data with varying sensor concentration and sample volume. Lipophilic solvatochromic dyes were also found to be much more effective FRET pairs than their hydrophilic counterparts, Fig. 5D.

With solvatochromic transducers there are now several unexplored interactions regarding the effects of surfactants on the indicator's local environment polarity. Wang *et al.* have noted that some of these sensors may be stabilised by the transducer itself due to its surfactant like nature,^36^ so there are opportunities to modulate sensor exchange equilibrium through surfactant choice rather than altering the transducer. Also, the stabilising ability of the solvatochromic transducer is likely to change as its density on the sensor surface changes in the course of the sensor response. Any modifications made to the headgroup to tune response range will consequently also alter particle stability. These factors may necessitate the need for particle stabilisers even when using anchored solvatochromic transducers. Additionally the optical response of hydrophilic solvatochromic dyes has been observed to have some dependence on the ionic strength of solution. This becomes pronounced at higher dye concentrations and ionic strengths where dyes are likely to encounter “Salting out” effects and the formation of aggregates. Although the internal organic phase of an ion selective optode can be highly controlled, the possible variance in the aqueous phase may be a point of concern when the charged head of lipophilic solvatochromic dyes transition.

There are also a number of unanswered questions when it comes to the implications of using solvatochromic dyes in particle-based systems that are raised by the earlier work with chromoionophore-based systems. The large shifts in p*K*_a_ values of chromoionophore transducers (Fig. 2) strongly suggests the existence of a polarity gradient within a particle that should also influence the optical signal of a solvatochromic transducer.

## Polyion sensing by controlling inner polarity of the nanoparticle ISOs

Recently, we have discovered a way to modulate the polarity of plasticizer-containing nanoparticles by doping them with dinonylnaphthalene sulfonate (DNNS). This may have important implications for the development of optical sensors for polyionic species that use DNNS as recognition element.

Two polyionic species of particular interest are heparin, a polyanionic glycosaminoglycan used as an anticoagulant in hospitals, and its polycationic antidote, protamine.^50,51^ Optical sensors for the detection of these two polyions have been described and successfully demonstrated in physiological samples. Previously reported sensors rely on typical ion exchange and ionic coextraction principles, see Fig. 1A and D.^10,52^ For example, the detection of protamine has been performed using a sensing film containing 2′,7′-dichlorofluorescein octadecyl ester (2′,7′-dichlorofluorescein with long alkyl chain) as chromoionophore. Protamine directly binds to 2′,7′-dichlorofluorescein octadecyl ester which is then deprotonated and changes absorption spectra. Modern ion exchange type protamine sensors commonly employ dinonylnaphthalenesulfonate (DNNS) as an ionophore which is more selective to protamine than the 2′,7′-dichlorofluorescein, ion exchanger/chromoionophore combination. Ion exchange of protamine and hydrogen ion arising from the deprotonation of a chromoionophore occurs for optical transduction, Fig. 1A. As for heparin detection, the very first heparin sensor has continued as the basis of modern optode mechanisms, specifically that based on coextraction of heparin and hydrogen ion into the film in which the chromoionophore is then protonated and changes its absorption.

Later, nanoparticle-based ISOs for polyion detection were introduced,^19,53^ very recently resulting in highly sensitive and rapidly responding protamine sensors. The improvement of the response time was especially notable because the response time of the first cast film ISO sensors was very slow owing to long equilibration times (slow diffusion).^54–58^ Nanoparticle sensors were found to equilibrate within seconds.^34,53^ The response time of membrane ISO sensors was recently improved by Hisamoto and coworkers by the use of very thin PVC membranes (*ca.* 140 nm).^59^ However, the sensing mechanism of such modern optode sensors makes use of the same ion exchange principle as the one employed in membrane-type sensors. Although the development of nanoscale optodes in the 2010s significantly improved response time and applicability to polyion detection,^60^ the fact remains that the sensing mechanism itself had not changed ever since the first development of polyion optode sensor in the 1990s.

Unfortunately, polyion-selective nanoscale sensors were failing in undiluted serum or plasma,^34,53,59^ requiring the use of diluted samples, while the membrane type sensors performed adequately in serum.^62^ Likely the most significant difference from cast film sensors is their dramatically larger surface to volume ratio. Considering that ionophore-based nanoparticles and thin membrane ISOs have shown promising performance in serum,^6,19,63,64^ these challenges appear to be specific to polyion ISOs. Indeed, studies on ion transfer voltammetry of polyions by Amemiya^55,57,65^ and spectroscopic studies by Meyerhoff^54^ suggest that such polyions may not only extract into the sensing phase but also interact with the sensing surface. It would seem plausible that a much larger surface area relative to sensing phase volume favors surface adsorption processes, which may make it difficult to realize optical sensors that are designed to rely on extraction principles.

**Fig. 6:**
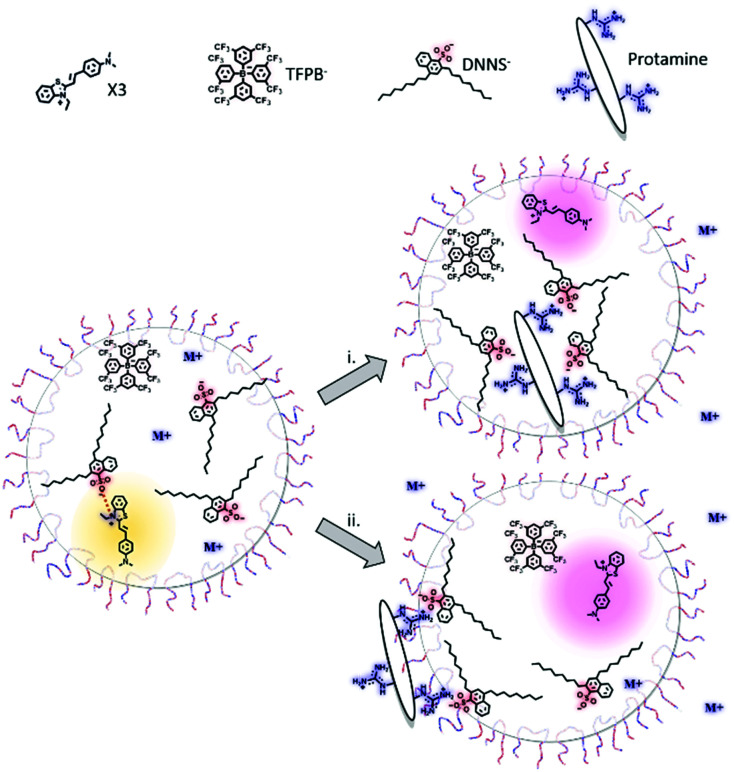
Reproduced from Soda *et al.*^61^ Proposed protamine (polycationic protein) detection mechanisms using a hyperpolarization-based sensor. The highly localised charge of DNNS strongly polarizes the solvatochromic dye, an interaction that is only interrupted by polyions that either localize in (i) or on (ii) the particle.

In our recent work, the negatively charged sulfonate group of DNNS was found to strongly polarize a solvatochromic dye in the nanoparticle bulk, more so than even an aqueous environment. Such an organic phase may be referred to as a hyper-polarizing organic phase. The strong interaction of DNNS with protamine^66^ is expected to disrupt the interaction with the dye. This then alters its local polarity and changes the absorption of the solvatochromic dye, providing an optical readout.^61^ The two proposed sensing mechanisms depend on whether protamine accumulates at the surface or is extracted and are illustrated in Fig. 6.

**Fig. 7:**
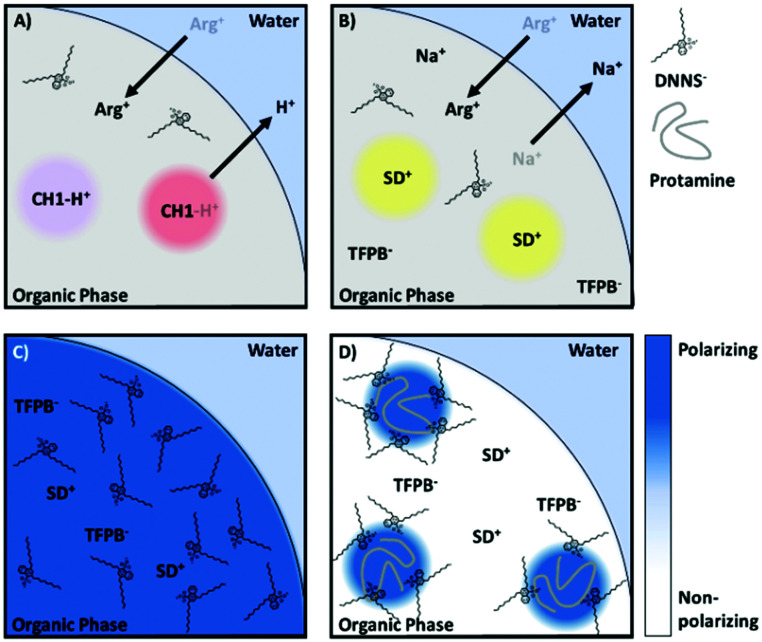
(A) Schematic showing non-selective response of sensor using direct optical transduction. (B) Sensor showing no optical response despite non-specific ion exchange. (C) A hyperpolarising organic phase with solvatochromic transducer. (D) Redistribution of DNNS following entry of polycation (protamine).

The intriguing feature of the above-mentioned hyper polarization-based sensor is the absence of ion exchange of the optical transducer, which contrasts with previously reported polyion sensor work. In contrast to direct ion exchange transduction shown in Fig. 7A, interference from any charged small molecule, even arginine, a main repeating amino acid of protamine, was completely absent with the hyperpolarization-based sensor, which finally enabled selective determination of protamine in serum and plasma. We believe the selectivity improvement is a result of the indirect signal transduction mechanism, whereby cooperative binding results in a highly partitioned distribution of the ligand (in this case DNNS) within the particle, a redistribution that only takes place following the introduction of a highly charged macromolecule, Fig. 7C–D. Also of note was the utility of F127, which increased the particle polarity gradients, which while generally detrimental, in this case increased the observed polarity shift while the new sensing mechanism maintained excellent selectivity regardless of the additional non-specific stabilisation of ions.

In our opinion there have been three significant of breakthroughs in optical polyion sensing, the first was the development of membrane ISOs capable of sensing polyion through ion exchange. The second is when the polyion sensing was demonstrated at the nanoscale and the dramatic improvements to response time were demonstrated. The third is the discovery of the hyperpolarization phenomenon which gave drastically improved sensor selectivity and finally enabled polyion sensing with nanoscale sensors in complex physiological samples.

Beyond polyion sensing some of the benefits of indirect transduction may be applied to other ion sensors and there is the possibility that ionophores with highly localized charges such as carbonate ionophores which have so far eluded applications in nanosensors may finally be realized. In addition some of the questions raised regarding possible aqueous phase variation effects on anchored solvatochromic dye signal are completely avoided by having a transducer that remains entirely in the organic phase.

## Conclusions an outlook

The advances discussed here promise advances across the field of optical sensing and beyond. The use of anchored solvatochromic dye, not only provides pH and volume independent sensor response but could also facilitate single ion pumps when coupled with photoswitchable ionophores. The application of non-partitioning surfactants goes a long way towards recovering the lost selectivity between films and particles often seen as a major disadvantage of particle-based sensors. It also raises questions regarding stabilization of sensors using surface accumulating anchored solvatochromic dyes and the effect of larger polymeric surfactants which could result in a third surface/surfactant phase. Indeed, significant work is still required to explore the possible interactions of lipophilic solvatochromic dyes operating through surface accumulation with a stabilizing surfactant. A third surfactant/polymer phase if highly controlled could provide a path to minimise signal deviations due to aqueous phase variance.

The behaviour of compounds within a sensing phase has in the past often been assumed as uniform, however, as discussed here, there is evidence of significant deviations especially in particle-based sensors. Such inhomogeneity may be induced by surfactants or polar ionophores. These internal sensing phase non-uniformities have actually proven very useful as they enable indirect transduction utilizing a hyperpolarizing organic phase. This transduction method has demonstrated significant improvements in polyion sensing in biological fluids, displaying no detectable interference from small ions. We believe indirect transduction could potentially provide benefits to other sensing systems in which the ionophore is highly polarising or when the ionophore–ion complex has some degree of unshielded charge.

## Conflicts of interest

There are no conflicts to declare.

## Supplementary Material

## References

[cit1] Bakker E., Bühlmann P., Pretsch E. (1997). Chem. Rev..

[cit2] Buhlmann P., Pretsch E., Bakker E. (1998). Chem. Rev..

[cit3] Arnold M. A., Meyerhoff M. E. (1984). Anal. Chem..

[cit4] Gantzer M. L. , Hemmes P. and Wong D., Eur. Pat., 0153641A2, 1985

[cit5] Seiler K., Simon W. (1992). Sens. Actuators, B.

[cit6] Soda Y., Citterio D., Bakker E. (2019). ACS Sens..

[cit7] Clark H. A., Hoyer M., Philbert M. A., Kopelman R. (1999). Anal. Chem..

[cit8] Müller B. J., Zhdanov A. V., Borisov S. M., Foley T., Okkelman I. A., Tsytsarev V., Tang Q., Erzurumlu R. S., Chen Y., Zhang H., Toncelli C., Klimant I., Papkovsky D. B., Dmitriev R. I. (2018). Adv. Funct. Mater..

[cit9] Koren K., Zieger S. E. (2021). ACS Sens..

[cit10] Wang E., Wang G., Ma L., Stivanello C. M., Lam S., Patel H. (1996). Anal. Chim. Acta.

[cit11] He X. M., Yam V. W. W. (2011). Org. Lett..

[cit12] Morf W. E., Seiler K., Lehmann B., Behringer C., Hartman K., Simon W. (1989). Pure Appl. Chem..

[cit13] Xie X., Gutiérrez A., Trofimov V., Szilagyi I., Soldati T., Bakker E. (2015). Anal. Chem..

[cit14] Huber C., Werner T., Krause C., Wolfbeis O. S., Leiner M. J. P. (1999). Anal. Chim. Acta.

[cit15] Charlton S. C., Fleming R. L., Zipp A. (1982). Clin. Chem..

[cit16] Charlton S. C. , Fleming R. L., Hemmes P. and Lau A. L. Y., US Pat., 4645744A, 1985

[cit17] Shortreed M., Bakker E., Kopelman R. (1996). Anal. Chem..

[cit18] Wang L., Bakker E. (2019). Chem. Commun..

[cit19] Xie X., Zhai J., Crespo G. A., Bakker E. (2014). Anal. Chem..

[cit20] Bakker E., Willer M., Pretsch E. (1993). Anal. Chim. Acta.

[cit21] Firooz A. R., Ensafi A. A., Hajyani Z. (2013). Sens. Actuators, B.

[cit22] Bakker E., Simon W. (1992). Anal. Chem..

[cit23] Bakker E., Meruva R. K., Pretsch E., Meyerhoff M. E. (1994). Anal. Chem..

[cit24] Shultz M. M., Stefanova O. K., Mokrov S. B., Mikhelson K. N. (2002). Anal. Chem..

[cit25] Mi Y. M., Bakker E. (1999). Anal. Chem..

[cit26] Lee H. J., Beriet C., Girault H. H. (1998). J. Electroanal. Chem..

[cit27] Bakker E., Willer M., Lerchi M., Seiler K., Pretsch E. (1994). Anal. Chem..

[cit28] Qin Y., Bakker E. (2002). Talanta.

[cit29] Bakker E., Lerchi M., Rosatzin T., Rusterholz B., Simon W. (1993). Anal. Chim. Acta.

[cit30] Lindner E., Rosatzin T., Jeney J., Cosofret V. V., Simon W., Buck R. P. (1993). J. Electroanal. Chem..

[cit31] Soda Y., Shibata H., Yamada K., Suzuki K., Citterio D. (2018). ACS Appl. Nano Mater..

[cit32] Soda Y., Bakker E. (2021). Anal. Chim. Acta.

[cit33] Xie X., Zhai J., Jarolímová Z., Bakker E. (2016). Anal. Chem..

[cit34] Xie X., Mistlberger G., Bakker E. (2013). Anal. Chem..

[cit35] Xie X., Bakker E. (2015). Anal. Chem..

[cit36] Wang L., Sadler S., Cao T., Xie X., Von Filseck J. M., Bakker E. (2019). Anal. Chem..

[cit37] Malinowska E., Manzoni A., Meyerhoff M. E. (1999). Anal. Chim. Acta.

[cit38] Espadas-Torre C., Bakker E., Barker S., Meyerhoff M. E. (1996). Anal. Chem..

[cit39] Soda Y., Gao W., Bosset J., Bakker E. (2020). Anal. Chem..

[cit40] Yan R., Luo X., Zhou J., Wang P., Yang Y., Qian X., Liu Y. (2021). Sens. Actuators, B.

[cit41] Li Y., Feng J., Huang Y., Qin Y., Jiang D., Chen H.-Y. (2020). Analyst.

[cit42] Zhang J., Chen Y., Fang D. (2020). J. Electroanal. Chem..

[cit43] Robinson K., Mao C., Bakker E. (2021). Anal. Chem..

[cit44] Xie X., Pawlak M., Tercier-Waeber M.-L., Bakker E. (2012). Anal. Chem..

[cit45] Wolfbeis O. S. (1995). Sens. Actuators, B.

[cit46] Huber C., Werner T., Krause C., Wolfbeis O. S. (1999). Analyst.

[cit47] Zhai J., Xie X., Bakker E. (2015). Anal. Chem..

[cit48] Wang L., Xie X., Zhai J., Bakker E. (2016). Chem. Commun..

[cit49] Xie X., Szilagyi I., Zhai J., Wang L., Bakker E. (2016). ACS Sens..

[cit50] Chargaff E., Olson K. B. (1937). J. Biol. Chem..

[cit51] Carr J., Silverman N. (1999). J. Cardiovasc. Surg..

[cit52] Wang E., Meyerhoff M. E., Yang V. C. (1995). Anal. Chem..

[cit53] Chen Q., Li X., Wang R., Zeng F., Zhai J., Xie X. (2019). Anal. Chem..

[cit54] Bell A. K., Höfler L., Meyerhoff M. E. (2012). Electroanalysis.

[cit55] Amemiya S., Yang X., Wazenegger T. L. (2003). J. Am. Chem. Soc..

[cit56] Garada M. B., Kabagambe B., Amemiya S. (2015). Anal. Chem..

[cit57] Yuan Y., Amemiya S. (2004). Anal. Chem..

[cit58] Fu B., Bakker E., Yun J. H., Yang V. C., Meyerhoff M. E. (1994). Anal. Chem..

[cit59] Mizuta T., Takai S., Nishihata T., Sueyoshi K., Endo T., Hisamoto H. (2020). Analyst.

[cit60] Xie X., Bakker E. (2015). Anal. Bioanal. Chem..

[cit61] Soda Y., Robinson K. J., Nussbaum R., Bakker E. (2021). Chem. Sci..

[cit62] Kim S. B., Kang T. Y., Cha G. S., Nam H. (2006). Anal. Chim. Acta.

[cit63] Du X., Zhai J., Zeng D., Chen F., Xie X. (2020). Sens. Actuators, B.

[cit64] Zhai J., Xie X., Cherubini T., Bakker E. (2017). ACS Sens..

[cit65] Garada M. B., Kabagambe B., Amemiya S. (2015). Anal. Chem..

[cit66] Boer C., Meesters M. I., Veerhoek D., Vonk A. B. A. (2018). Br. J. Anaesth..

